# AI HeartBot to Increase Women’s Awareness and Knowledge of Heart Attacks: Nonrandomized, Quasi-Experimental Study

**DOI:** 10.2196/80407

**Published:** 2025-10-15

**Authors:** Yoshimi Fukuoka, Diane Dagyong Kim, Jingwen Zhang, Thomas J Hoffmann, Holli A DeVon, Kenji Sagae

**Affiliations:** 1 Department of Physiological Nursing, University of California, San Francisco, 521 Parnassus Ave, San Francisco, CA, 94143, United States, 1 415-476-8419; 2 Department of Communication, University of California, Davis, Davis, CA, United States; 3 Department of Epidemiology and Biostatistics, University of California, San Francisco, San Francisco, CA, United States; 4 University of California, Los Angeles, Los Angeles, CA, United States; 5 Department of Linguistics, University of California, Davis, Davis, CA, United States

**Keywords:** artificial intelligence, AI, chatbot, natural language processing, heart disease, heart attack symptoms, women, conversational agent

## Abstract

**Background:**

Heart disease remains a leading cause of death for women in the United States, but awareness and knowledge about it are declining. Artificial intelligence (AI) chatbots have great potential to educate women.

**Objective:**

This study aimed to evaluate the potential efficacy of HeartBot to increase women’s awareness and knowledge of heart attack symptoms and care-seeking behavior.

**Methods:**

In this nonrandomized pilot, quasi-experimental study, 92 women aged ≥25 years without a history of heart disease completed the HeartBot interaction via SMS text messaging. The study was remotely conducted from October 2023 to January 2024. HeartBot, a fully automated AI chatbot, covered 15 topics of heart attack awareness, knowledge, symptoms, and care seeking in a single session. The mean length of the HeartBot interaction was 13.0 (SD 7.80) minutes. The primary outcomes consist of four questions: (1) recognizing signs and symptoms of a heart attack, (2) telling the difference between the signs and symptoms of a heart attack, (3) calling an ambulance or dialing 911 when experiencing heart attack symptoms, and (4) getting to an emergency room within 60 minutes after the onset of symptoms of a heart attack. Women were asked to answer the 4 questions before and after the HeartBot interaction on a scale of 1 to 4, with a higher score indicating higher levels of awareness and knowledge of heart attack risks and symptoms.

**Results:**

The mean age of the sample was 45.9 (SD 11.9) years. In total, 59.8% (55/92) of the sample identified as belonging to racial or ethnic minority groups. The mean length of the HeartBot interaction was 13.0 (SD 7.80) minutes. In ordinal logistic regression models, women showed significant improvements across the 4 self-reported outcomes (ie, heart attack symptoms and calling 911) even after controlling for potential confounding factors (outcome 1: adjusted odds ratio [aOR] 7.10, 95% CI 3.52‐13.16; outcome 2: aOR 5.47, 95% CI 2.77‐10.78; outcome 3: aOR 5.75, 95% CI 2.86‐11.59; and outcome 4: aOR 2.85, 95% CI 1.54‐5.25; *P*<.001 for all 4 outcomes).

**Conclusions:**

HeartBot led to a substantial increase in awareness and knowledge of heart attack risks and symptoms in women. These findings suggest that HeartBot is a promising approach to improving heart health education. A randomized controlled trial of HeartBot is warranted to establish its efficacy and safety for the clinical setting.

## Introduction

### Background

Artificial intelligence (AI) chatbots using natural language processing and machine learning facilitate natural human-machine conversations. In recent years, AI chatbots have gained significant popularity in health care and research, and researchers have investigated their efficacy across health domains. AI chatbots have shown potential to improve mental health [1-3] and other chronic illnesses [4] and to promote healthy lifestyles and self-care behaviors [5-10]. However, research on the use of chatbots in cardiovascular health is still in its infancy.

Heart disease continues to be the leading cause of mortality and morbidity for women in the United States [11]. More than 60 million women in the United States are living with heart disease [12]. Over the past 2 decades, several awareness campaigns, such as *Go Red for Women* [13] by the American Heart Association, have been conducted to educate the public and women regarding heart disease. Despite these large-scale public health campaigns, awareness of heart disease as the leading cause of death among women declined from 65% in 2009 to 44% in 2019 [14]. The greatest declines in awareness were observed among Hispanic and Black women as well as younger women [15-17].

### Objectives

We therefore need a new approach to increase knowledge and awareness of heart disease in women. We recently developed and tested an AI chatbot (hereafter referred to as “HeartBot”) in a series of studies to achieve this goal. Given the rapid advancement of AI technologies and the increasing prevalence of smartphone ownership [18], HeartBot could have significant advantages over traditional public health campaigns, expanding reach and delivering personalized communication. Therefore, this study aims to evaluate the potential efficacy of a fully automated AI HeartBot in increasing women’s awareness and knowledge of heart attack symptoms and care-seeking behavior.

## Methods

### Study Design and Sample

We remotely conducted a pilot, quasi-experimental study with 92 participants using the user-centered design approach [19]. Eligibility criteria were women aged ≥25 years, no self-reported cognitive impairment or history of heart disease or stroke, not a health care professional or student, not working in the health care field, living in the United States, possessed a cell phone with the ability to send and receive text messages, and had internet access. Participants were recruited via social media (ie, Facebook and Instagram [Meta]). The study was conducted from October 2023 to January 2024.

### Description of the HeartBot Platform

**Figure 1.**
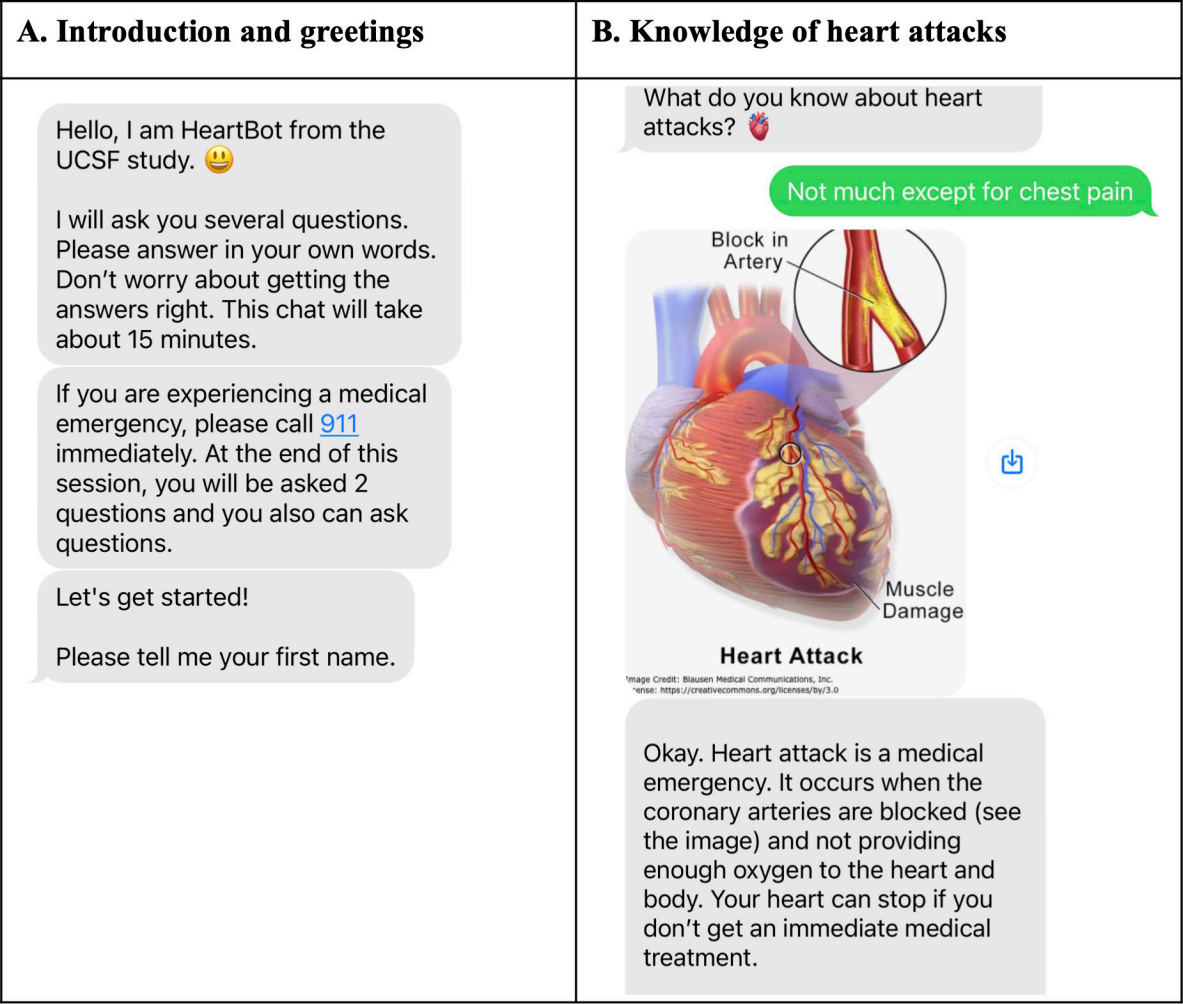
Screenshots of the HeartBot conversation. The text in gray bubbles represents messages sent by HeartBot, while that in the green bubble represents the responses from the users. UCSF: University of California, San Francisco.

Table 1 provides an overview of the HeartBot conversation content, and Figure 1 shows the screenshots of the HeartBot conversation. HeartBot conversations occurred over SMS text messaging and started with a brief introduction, informing the user about what to expect in the conversation (Figure 1A). Within 1 conversational episode, HeartBot conversed with participants on 17 content modules, covering topics such as symptoms, risk factors, and treatment of heart attacks. To prioritize participant safety, the introduction message included the following medical emergency notice: “If you are experiencing a medical emergency, please call 911 immediately.” The messages sent by HeartBot (Table 1) were developed by cardiovascular experts based on the latest guidelines and evidence to ensure full control over the content presented to participants and to minimize the risk of having the system dispense false or misleading information. In addition, we incorporated personalization and empathic responses—key communication features designed to enhance participants’ experience and engagement [20]. Finally, to ensure readability, the content sent by HeartBot (Table 1) was evaluated using Flesch-Kincaid readability metrics. This analysis yielded a Flesch reading ease score of 69 and a Flesch-Kincaid grade level of 6.2, indicating that the language used was accessible and comprehensible to a broad audience.

**Table 1.** Overview of the message sequence and conversation content delivered by HeartBot, an artificial intelligence chatbot designed to increase women’s awareness and knowledge of heart attack risks and symptoms.^a^

Message order	Topics
1	Introduction and greetings
2	Participants’ name retrieval
3	Knowledge of heart attacks
4	Symptoms of heart attacks
5	Leading cause of death for women in the United States
6	Gender factors for heart attacks
7	First action when experiencing symptoms of a heart attack
8	Importance of calling 911
9	Time to seek medical help
10	Treatment of heart attacks
11	Action plans while waiting for 911
12	Risk factors for heart disease
13	Female-specific risk factors for heart disease
14	Racial and ethnic differences in women’s heart disease risk
15	Multiple-choice questions
16	Further questions to ask HeartBot
17	Acknowledgment and conclusion of the conversation

a Within a single conversational episode, HeartBot delivers 17 sequential modules developed by cardiovascular experts based on current clinical guidelines. Messages were evaluated for readability using Flesch-Kincaid metrics (reading ease score=69; grade level=6.2).

The current version of HeartBot, deployed to research participants, is based on an established framework that has been used in a large number of chatbots across various domains over several years. HeartBot was built using the Google Dialogflow CX platform connected to Twilio for input and output over SMS text messaging. Dialogflow CX supports the development and deployment of chatbots based on the intents and entities paradigm [21]. Every user input is categorized as one of a finite number of intents (eg, greeting, correct response, and request for help), and chatbot actions, including what the chatbot says next, are mapped directly to these intents. In HeartBot specifically, this mapping was implemented using a bidirectional encoder representations from transformers neural language model [22], which is based on the transformer architecture [23]; this approach leverages a powerful data-driven method for robustly identifying user intents expressed in free-form text messages. Within each participant’s utterance, one or more entities may be mentioned. Entities are words or phrases that express specific instances of a class. For example, “high blood pressure” expresses a specific instance of the class *risk factors*. Authoring a chatbot in Dialogflow CX involves the following: (1) defining the intents that the user may express when interacting with the chatbot and which entities the chatbot should recognize within user utterances; (2) listing examples of how these intents and entities can be expressed; and (3) defining the chatbot actions that should be mapped to each intent and, optionally, to mentions of an entity. While the use of a bidirectional encoder representations from transformers language model allows the system to handle natural language input flexibly and robustly, the chatbot’s behavior and responses are limited to content authored specifically for HeartBot conversations. Because each piece of the interaction is encoded directly and explicitly into the chatbot, the resulting interactions are rigid and predictable, varying in response to user utterances only in the ways intended by the system’s designers. On the one hand, this ensures a great level of control over what the chatbot can do, and on the other hand, the chatbot has limited flexibility and lacks any conversational ability beyond its authored content. In addition, the design of HeartBot follows a strict system initiative philosophy: every step of the interaction between the system and the user is initiated by the system. This is in contrast to the user-initiative design embodied in current generative AI chatbots, such as ChatGPT, where the system mainly reacts to or responds to user utterances, and the user is primarily responsible for directing the conversation. HeartBot asks specific questions, and the user responds to them. While HeartBot’s utterances vary based on the user’s responses, for example, by confirming correct responses or offering gentle corrections, the user has very limited control over how the interaction unfolds, and the system is in control of the conversation.

HeartBot’s content library and the inventory of user intents it can handle were designed and refined based on more than 171 interactions where a research staff with domain expertise played the role of the system. These interactions took place over text messages in the same way as the HeartBot interactions, except that participants were not told they were interacting with HeartBot and had no reason to believe their conversational partner was a chatbot. The research staff conducted the conversation initially designed for HeartBot in each of these interactions. The initial version of HeartBot was based on these human-human interactions and was further refined through initial system testing, resulting in the version of HeartBot used to collect the data presented here.

### Procedures

Potential participants interested in the study were asked to complete an online screening survey on Research Electronic Data Capture (REDCap; Vanderbilt University) to determine eligibility. Research staff sent an electronic consent (e-consent) form to those meeting all eligibility criteria. Participants who signed the consent form received a baseline REDCap survey. Upon completing the baseline survey, research staff provided instructions with a specific phone number for participants to send SMS text messages to and on how to initiate a conversation with HeartBot. For participants’ safety, research staff closely monitored the participants’ responses during the HeartBot conversations through a back-end data monitoring platform. Research staff sent an online postsurvey link to the participants 4 to 6 weeks after the HearBot interaction. Participants who completed all study requirements received a US $20 Amazon e-gift card.

### Measures

We used a previously validated set of questions to assess the potential efficacy of HeartBot in increasing the awareness and knowledge of heart attack risks and symptoms [24,25]. These items have been applied in earlier studies with women from varied backgrounds to support their relevance across diverse populations [26-28]. Participants answered four questions before and after interacting with HeartBot, using a 4-point scale, in which 1 indicated “not sure” and 4 indicated “sure”: (1) How sure are you that you could recognize the signs and symptoms of a heart attack in yourself?; (2) How sure are you that you could tell the difference between the signs or symptoms of a heart attack and other medical problems?; (3) How sure are you that you could call an ambulance or dial 911 if you thought you were having a heart attack?; and (4) How sure are you that you could get to an emergency room within 60 minutes after onset of your symptoms of a heart attack? Higher scores indicate a better awareness and knowledge of heart attack risks and symptoms.

To understand how participants engage with HeartBot and identify areas of improvement for HeartBot, we examined participants’ evaluations of HeartBot using the *Effectiveness Scale*, a semantic-differential scale originally developed based on previous literature [29,30]. It consists of 5 pairs of opposite adjectives (effective vs. ineffective, helpful vs. unhelpful, beneficial vs. not beneficial, adequate vs. not adequate, supportive vs. not supportive), and each pair is scored on a 7-point Likert scale. 1 indicated the negative pole (eg, “ineffective”) and 7 indicated the positive pole (eg, “effective”). Cronbach’s alpha for this sample was 0.94. In addition, the impression of HeartBot was assessed by using the *Anthropomorphism Scale* [31] consisting of 5 pairs of opposite adjectives (fake vs natural, machine-like vs humanlike, unconscious vs conscious, artificial vs lifelike, rigid vs adaptive). Each pair was rated on a 7-point Likert scale, 1 being the first adjective in the pair (eg, “fake”) and 7 being the second adjective (eg, “natural”). The scores for each pair in the *Effectiveness Scale* and *Anthropomorphism Scale* were summed and averaged to create mean composite scores. In this current sample, both measures had excellent reliability (Cronbach α 0.94 and 0.91, respectively).

### Statistical Analysis

We conducted a descriptive analysis to summarize sample characteristics, including sociodemographic characteristics, cardiovascular risks, and usability outcomes. To compare pre- and postsurvey results, we used ANOVA for continuous variables and chi-square tests for categorical variables to assess distributional differences. To determine whether participants’ responses to awareness and knowledge questions significantly changed after engaging with HeartBot, we first used Wilcoxon signed-rank tests. Then, to test for differences in pre- to post-intervention awareness and knowledge of heart attack risks and symptoms as outcome responses, we fit an ordinal mixed effects logistic regression model using the R (version 4.1.0; R Foundation for Statistical Computing) [32] package *ordinal* v2022.11.16 [33], adjusting for fixed effects of White (vs non-White), age, education, income, family history, past chatbot use, mean text message effectiveness, mean HeartBot impression score, word count, conversation duration, and whether the individual thought they were texting an AI agent, as well as a random effect for individual, across the 92 participants with outcomes measured at the second time point. As a sensitivity analysis, although this model appeared to fit the data well, we also conducted a backward stepwise regression analysis (*P*<.05; resulting in 2‐5 covariates per outcome) to ensure the model was not overparameterized. Additional sensitivity analyses included using all participants through multiple imputation for missing outcomes (instead of just imputing missing covariates), as well as a complete case analysis (2 of the 92 participants were missing income data). We also attempted a sensitivity analysis by fitting a mixed-effects multinomial logistic regression model using the generalized structural equations command in Stata (version 16.1; StataCorp LLC) [34]. However, these models did not converge, likely due to the limited sample size and increased number of parameters to estimate compared to an ordinal logistic regression model. To appropriately handle missing data, we used multiple imputation with chained equations with 100 imputations, combined via the usual Rubin rules, using a logistic regression model for dichotomous variables and perfect mean matching for continuous variables, using the R package *mice* v3.16.0 [35]; 2 random seeds were run to ensure stability of results. All analyses used 2-tailed tests, and statistical significance was evaluated at *P*<.05. The awareness and knowledge of heart attack risks and symptoms questions were coded as time-dependent variables at baseline and post-HeartBot interaction.

### Ethical Considerations

This study was conducted in accordance with the ethical standards outlined in the Declaration of Helsinki. Institutional review board approval was obtained from the University of California, San Francisco (approval: 23‐39793). All participants provided written informed consent before study enrollment. Participation was voluntary, and participants were informed that they could withdraw at any time without penalty. All collected data were deidentified before analysis, and no personally identifiable information was retained. Data were stored on secure, password-protected servers accessible only to the research team. Participants who completed all study requirements received a US $20 Amazon e-gift card as compensation.

## Results

### Baseline Sample Characteristics

A total of 104 women completed the online baseline survey. Of these 104 women, 5 (4.8%) did not start the HeartBot interaction, and 7 (6.7%) did not complete the post-REDCap online survey. The sample characteristics of 12 women who did not complete the study did not differ from those who completed the study (*P*>.05). Table 2 presents the baseline characteristics of 92 women who completed all study requirements. The mean age was 45.9 (SD 11.9; range: 26-70) years. Out of 92 participants, 55 (59.8%) identified as belonging to racial or ethnic minority groups; 53 (57.6%) had used a chatbot such as Alexa, Google Assistant, and Siri, at least once in the past 30 days; and 13 (14.1%) reported a family history of heart attack.

**Table 2.** Baseline sociodemographic characteristics and cardiovascular risk factors of study participants (N=92).

Sociodemographic characteristics	Value
Age (years), mean (SD)	45.9 (11.9)
Race or ethnicity, n (%)	
American Indian	1 (1.1)
Asian	6 (6.5)
Black (non-Hispanic)	22 (23.9)
Hispanic	19 (20.7)
Native Hawaiian	2 (2.2)
White (non-Hispanic)	37 (40.2)
More than 1 race or ethnicity	5 (5.4)
Education, n (%)	
Less than high school or did not complete college	26 (28.3)
Completed college or graduate school	66 (71.7)
Household income (US $), n (%)	
Less than 40,000	21 (22.9)
40,001-75,000	30 (32.6)
Greater than 75,000	39 (42.4)
Do not know or decline to answer	2 (2.2)
Marital status, n (%)	
Never married	21 (22.8)
Currently married or cohabitating	59 (64.1)
Divorced or widowed	12 (13)
Employment status, n (%)	
Full time or part time	56 (60.9)
Unemployed, homemaker, or student	17 (18.5)
Retired, disabled, or other	19 (20.7)
Chatbot use (eg, Amazon’s Alexa, Google Assistant, Siri, and Facebook Messenger bot) in the past 30 days, n (%)	
Yes	53 (57.6)
No	39 (42.4)
Self-reported cardiovascular risks, mean (SD)	
BMI (kg/m^2^)	29.5 (7.1)
Smoked at least 1 cigarette in the past 30 days, n (%)	
Yes	14 (15.2)
No	78 (84.8)
Blood pressure medication, n (%)	
Yes	25 (27.2)
No or do not know	67 (72.8)
Cholesterol medication, n (%)	
Yes	29 (31.5)
No or do not know	63 (68.5)
Diabetes medication, n (%)	
Yes	12 (13)
No or do not know	80 (85.9)
Family history of heart disease or stroke, n (%)	
Yes	13 (14.1)
No or do not know	79 (85.9)

### HeartBot Interaction

The mean and median duration of HeartBot interactions were 13.0 (SD 7.8) and 10.6 (IQR 8.5-13.9) minutes, respectively (Table 3). In addition, a post hoc analysis of HeartBot interaction logs showed that 11% (10/92) conversations had a minor technical issue in which participants were prompted to provide their name twice at the start of the session. This glitch did not interfere with delivering or completing the conversation, and all affected participants successfully completed the HeartBot session. At the end of the HeartBot conversation, 29% (27/92) of the participants submitted at least 1 question to HeartBot. The top two questions that the participants asked were regarding (1) heart disease risks and prevention risk reduction and (2) signs and symptoms of a heart attack and care-seeking behaviors during a heart attack. The participants rated HeartBot as highly effective (mean 5.7, SD 1.2) and as human-like and natural (mean 5.2, SD 1.2), with scores ranging from 1 to 7.

**Table 3.** HeartBot interaction (N=92).^a^

Interaction metrics	Value
Total word count, mean (SD)	890.1 (78.7)
Duration of HeartBot conversation (minutes), mean (SD)	13.0 (7.80)
Number of questions participants asked, n (%)	
No question	65 (70.7)
At least 1 question	27 (29.3)
HeartBot effectiveness, mean (SD)	5.7 (1.2)
HeartBot impression, mean (SD)	5.2 (1.2)
“Overall, how would you rate the conversations with your texting partner?” n (%)	
Unnatural or very unnatural	5 (5.4)
Neutral	33 (35.9)
Natural or very natural	54 (58.7)
“Overall, how would you rate the messages you received?” n (%)	
Incoherent or very incoherent	0 (0)
Neutral	23 (25.0)
Coherent or very coherent	69 (75.0)
“Do you think you texted a human or an AI^b^ chatbot during your conversation?” n (%)	
Human	31 (33.7)
AI chatbot	61 (66.3)
”Have you ever heard or read about AI?” n (%)	
I would consider myself an expert in that field	5 (5.4)
I could explain well and what AI is about	27 (29.3)
I know somehow what AI is	41 (44.6)
Yes, but I do not know exactly what AI is	16 (17.4)
No	3 (3.3)
“How positive or negative do you feel about the use of AI in health care?” n (%)	
Negative or very negative	13 (14.1)
Neutral	44 (47.8)
Positive or very positive	35 (38)
“The use of AI will result in better health care,” n (%)	
Strongly disagree or disagree	17 (18.5)
Neither agree nor disagree	30 (32.6)
Agree or strongly agree	45 (48.9)
“The use of AI will result in better health outcomes,” n (%)	
Disagree or strongly disagree	16 (17.4)
Neither agree nor disagree	35 (38.0)
Agree or strongly agree	41 (44.6)
“AI may help me reduce my risk of heart disease,” n (%)	
Disagree or strongly disagree	12 (13.0)
Neither agree nor disagree	39 (42.4)
Agree or strongly agree	41 (44.6)

a Participant interaction metrics, ratings of HeartBot conversations, and attitudes toward artificial intelligence (AI): measures include conversation length, number of participant-initiated questions, perceived effectiveness, naturalness, coherence, identification of the agent as a chatbot or human, prior AI awareness, and attitudes toward the use of AI in health care and for heart disease prevention.

b AI: artificial intelligence.

### Changes in Awareness and Knowledge of Heart Attack Risks and Symptoms

Table 4 shows the results of the Wilcoxon signed-rank tests for the changes in responses to awareness and knowledge of heart attack risks and symptoms questions between the pre- and post-HeartBot interaction. Participants reported significantly higher awareness and knowledge of heart attack risks and symptoms across all 4 outcome responses between the baseline and the post-HeartBot interaction (*P*<.05).

Table 5 shows the results of the ordinal mixed effects regression models for each awareness and knowledge of heart attack risks and symptoms question. Even after controlling for potential confounders (Table 5; the full model and sensitivity analysis are provided in Multimedia Appendix 1), the HeartBot interaction was significantly associated with improvements in awareness and knowledge of heart attack risks and symptoms, specifically on (1) recognizing the signs and symptoms of a heart attack response (adjusted odds ratio [aOR] 7.10, 95% CI 3.56‐14.15; *P*<.001), (2) telling the difference between the signs or symptoms of a heart attack (aOR 5.47, 95% CI 2.77‐10.78; *P*<.001), (3) calling an ambulance or dialing 911 during a heart attack (aOR 5.75, 95% CI 2.86‐11.59; *P*<.001), and (4) getting to an emergency room within 60 minutes after onset of symptoms (aOR 2.85, 95% CI 1.54‐5.25; *P*<.001). Results were very similar across all sensitivity analyses (Table S1 in Multimedia Appendix 1).

**Table 4.** Changes in participants’ knowledge and awareness of symptoms and responses to heart attack between pre- and post-HeartBot interaction (N=92).

		Pre-HeartBot interaction, n (%)	Post-HeartBot interaction, n (%)	*P* value^a^
How sure are you that you could recognize the signs and symptoms of a heart attack in yourself? (please select a number from 1‐4)				<.001
	Not sure	24 (26.1)	3 (3.3)	
	Somewhat not sure	32 (34.8)	28 (30.4)	
	Somewhat sure	33 (35.9)	40 (43.5)	
	Sure	3 (3.3)	21 (22.8)	
How sure are you that you could tell the difference between the signs or symptoms of a heart attack and other medical problems? (please select a number from 1‐4)				<.001
	Not sure	28 (30.4)	8 (8.7)	
	Somewhat not sure	38 (41.3)	35 (38)	
	Somewhat sure	24 (26.1)	40 (43.5)	
	Sure	2 (2.2)	9 (9.8)	
How sure are you that you could call an ambulance or dial 911 if you thought you were having a heart attack? (please select a number from 1‐4)				.02
	Not sure	13 (14.1)	3 (3.3)	
	Somewhat not sure	20 (21.7)	13 (14.1)	
	Somewhat sure	32 (34.8)	20 (21.7)	
	Sure	27 (29.3)	56 (60.9)	
How sure are you that you could get to an emergency room within 60 min after the onset of your symptoms of a heart attack? (please select a number from 1‐4)				<.001
	Not sure	17 (18.5)	6 (6.5)	
	Somewhat not sure	17 (18.5)	12 (13)	
	Somewhat sure	29 (31.5)	31 (33.7)	
	Sure	29 (31.5)	43 (46.7)	

a Wilcoxon signed-rank test.

**Table 5.** Ordinal logistic regression models for heart attack questions.

Outcome	Unadjusted OR^a^		Adjusted OR^b^	
	OR (95% CI)	*P* value	OR (95% CI)	*P* value
Recognize the signs and symptoms of a heart attack	7.20 (3.63‐14.27)	<.001	7.10 (3.56‐14.15)	<.001
Tell the difference between the signs or symptoms of a heart attack and other medical problems	5.18 (2.66‐10.09)	<.001	5.47 (2.77‐10.78)	<.001
Call an ambulance or dial 911	5.81 (2.89‐11.68)	<.001	5.75 (2.86‐11.59)	<.001
Get to an emergency room within 60 minutes	2.94 (1.61‐5.38)	<.001	2.85 (1.54‐5.25)	<.001

a OR: odds ratio.

b Models are adjusted for the fixed effects of race (White vs non-White), age, education, income, family history, prior chatbot use, mean text message effectiveness score, mean HeartBot impression score, word count, conversation duration, and participants’ perception of texting an artificial intelligence agent, as well as a random effect for individual.

## Discussion

### Principal Findings

This study is among the first to evaluate a fully automated AI chatbot aiming to increase awareness and knowledge about heart attacks among women. All participants who started the HeartBot conversation completed it, and none left the conversation. We posit that the 3 main key factors contributed to HeartBot’s success. First, HeartBot delivered a brief conversational intervention (median 10.33 min), and participants could initiate the intervention anywhere and at any time. This design dramatically reduces participant burden and ensures a potential for large-scale dissemination and effectiveness. In addition, the HeartBot conversation was delivered via SMS text messaging and did not require Wi-Fi access or a smartphone. Nearly all Americans (98%) own a mobile phone [36], and using SMS text messaging is an effective way to reach minority populations and ensure intervention equity. Second, HeartBot incorporated personalization and empathetic responses that are known to increase participants’ engagement [11-13] during conversations. Addressing users by name within conversations could have fostered a sense of individual relevance and strengthened a positive evaluation of HeartBot. Third, the content of HeartBot was developed from scientific evidence and guidelines and used simple nontechnical language with images, ensuring that the educational content was easily understandable and accessible to individuals with low health literacy levels.

### Comparison With Prior Work

Previous Hispanic and Black women, and younger age groups, have shown a significant decline in awareness of heart disease as the leading cause of death among women [14-17]. The latest study reported that the overall awareness dropped from 65% in 2009 to 44% in 2019, with the steepest declines among Hispanic women (28.9%), non-Hispanic Black women (28.1%), and women aged 25 to 34 years (41.3%) [14]. To address this concern, we ensured that women with racial or ethnic minority backgrounds were well represented in this study sample, and the HeartBot included a content module specifically discussing the increased risk of heart attack in Hispanic and Black women [37,38]. HeartBot provided unbiased interactions with all participants, reducing potential biases that might arise from human facilitators in traditional in-person interventions, such as implicit judgments [39,40]. The HeartBot intervention appears to work regardless of age and race or ethnicity. To our knowledge, this is the first AI-driven chatbot intervention specifically designed to increase women’s awareness and knowledge of heart disease. The greatest advantage of the HeartBot intervention over conventional public heart attack awareness campaigns is its ability to promote active learning through personalized and SMS-based dialogue. Traditional heart attack campaigns face several challenges, such as passive information delivery, limited personalization and interactivity, and dissemination gaps. In contrast, personalized chatbots can foster active learning by prompting users to engage in feedback retrieval, self-reflection, and goal setting during interactions [41,42]. We believed that the inclusion of a follow-up quiz reviewing key content at the end of the session further enhanced active learning throughout the session.

Overall, the findings of this HeartBot trial hold great promise. Although we acknowledge that generative AI models are improving at a rapid pace, we think deploying a hybrid chatbot, which combines data-driven identification of user intents with scientifically vetted conversational content, is essential to prioritize participants’ safety and accuracy of information delivery. Given the purpose of the intervention to increase awareness and knowledge of heart attacks, the current design, which combines a structured content flow with personalized conversational strategies, may be optimal.

### Limitations

Several limitations of this study must be acknowledged. First, because this study was not a randomized controlled trial (RCT), causal relationships and definitive efficacy cannot be established. However, we adjusted potential confounding factors in our analysis. Second, relying on a convenience sample of women may introduce selection bias. In addition, only participants who had at least moderate digital literacy, were comfortable with mobile technology, and were interested in their own health might be enrolled in this study, which could have influenced both engagement with the chatbot and the outcomes. To mitigate these section biases, we enrolled a wide age range (ie, 26-70 y) and a diverse sample of women (ie, 55/92, 60% belonging to racial or ethnic minority groups), as well as designed the study to not require Wi-Fi access (SMS text messaging only). Finally, the primary outcomes were assessed using self-reported measures of awareness and knowledge of heart attack risks and symptoms over a short period; behavioral changes were not assessed. Social desirability bias may have led some participants to overreport positive impressions or improvements in knowledge following the HeartBot interaction.

### Future Direction

A full-scale RCT is warranted to examine the effectiveness of the HeartBot intervention in a large, racially and ethnically diverse sample. Future research also needs to aim to assess whether increased awareness and knowledge will lead to early access to care when women are experiencing heart attack symptoms. The framework of participants’ engagement and safety metrics must be developed to demonstrate use for clinical practice. Finally, future research should explore the duration of the effects of new awareness and knowledge and whether they lead to desired behaviors in an RCT. To sustain the awareness and knowledge of heart attacks, the HeartBot intervention may require multiple sessions, and the dosage and content of the intervention may need further adjustment.

### Conclusions

Interaction with HeartBot was associated with increased awareness and knowledge of heart attack risks and symptoms in a national sample of US women. These findings suggest that AI chatbot–based interventions may be a promising approach to improve women’s knowledge and awareness of heart attack in the United States. An RCT of HeartBot is warranted to establish its efficacy and safety before implementation in clinical settings.

## Supplementary material

10.2196/80407 Multimedia Appendix 1 Full regression results for outcome questions. The “Adjusted OR” reflects results from the subset of participants with measured outcomes (n=92), as reported in the paper. The first sensitivity analysis includes the full dataset (n=104), and the second is a complete case analysis (n=90; 2 participants were missing income data). The remaining sensitivity analyses correspond to these 3 models but use backward stepwise regression. Cells show odds ratio (95% CI). Outcomes were as follows: (1) recognizing the signs and symptoms of a heart attack, (2) distinguishing the signs or symptoms of a heart attack from other medical problems, (3) calling an ambulance or dialing 911, and (4) reaching an emergency room within 60 minutes.

## Abbreviations:

AI: artificial intelligence
aOR: adjusted odds ratio
RCT: randomized controlled trial
REDCap: Research Electronic Data Capture
